# Dietary Glycerol Monolaurate Enhances Growth and Immune Function in Calves via Hepatic Immunometabolic Reprogramming

**DOI:** 10.3390/vetsci13060572

**Published:** 2026-06-10

**Authors:** Ao Dong, Xitong Guan, Yuxuan Cao, Jiahui Cao, Yuxuan Yan, Yueyang Zhao, Xiangfang Tang, Yufan Zhao, Yonggen Zhang, Shunjin Jiang, Yang Li

**Affiliations:** 1 College of Animal Science and Technology, Northeast Agricultural University, Harbin 150030, China; 18745769350@163.com (A.D.); gxt20010827@163.com (X.G.); caoyuyuxuan@126.com (Y.C.); 17612845180@163.com (J.C.); 18800496696@163.com (Y.Y.); 18246893818@163.com (Y.Z.); zyf19994268@163.com (Y.Z.); zhangyonggen@neau.edu.cn (Y.Z.); 2 State Key Laboratory of Animal Nutrition, Institute of Animal Science, Chinese Academy of Agricultural Sciences, Beijing 100193, China; tangxiangfang@caas.cn; 3 Guangdong Rongda Biology Co., Ltd., Qingyuan 511510, China; shunjin2000@sina.com

**Keywords:** glycerol monolaurate, early health of calves, growth performance, liver transcriptome, lipid metabolism

## Abstract

Early-life nutrition is critical for the development and health of dairy calves, necessitating alternatives to in-feed antibiotics. This study investigated whether dietary glycerol monolaurate (GML) enhances growth performance, reduces diarrhea incidence, improves systemic antioxidant and immune status, and modulates hepatic immunometabolic function in calves. Twenty-four Holstein bull calves (7 ± 0.5 d of age) were randomly assigned by body weight and age to a control group or a GML-supplemented group, both fed milk replacer with starter feed provided throughout the 45-day trial. Calves in the GML group received GML at a dosage of 100 mg/kg of body weight, mixed into the milk replacer prior to feeding. Calves in the GML group had significantly greater final body weight, average daily gain, and starter intake during the latter period (d 23–45) compared with the control group. GML supplementation also significantly reduced the incidence of diarrhea and fever, alongside lower fecal scores and fewer antibiotic treatments. Plasma analysis revealed enhanced antioxidant capacity, as indicated by increased total antioxidant capacity and glutathione peroxidase, along with an improved immune profile characterized by elevated immunoglobulin G and reduced interleukin-2. Transcriptomic analysis of the liver showed that GML upregulated genes and pathways related to innate antiviral immunity, such as radical S-adenosyl methionine domain containing 2, interferon-stimulated gene 15, and MX dynamin like GTPase 1. Lipidomics further indicated that GML induced a targeted remodeling of hepatic lipids, including increased diacylglycerols and triacylglycerols and decreased specific phospholipids and sphingolipids, suggesting a metabolic shift supportive of immune activation and inflammatory control. In conclusion, dietary GML enhances growth and health in suckling calves, which is mediated through a coordinated immunometabolic reprogramming in the liver. GML represents a promising functional fat additive for sustainable calf rearing.

## Simple Summary

Young calves are highly vulnerable to digestive disorders and infections, especially during early life when their immune and digestive systems are still developing. This study evaluated whether adding glycerol monolaurate (GML), a natural fatty acid compound found in coconut oil, to milk replacer could improve calf health and growth. Calves receiving GML grew faster, had better feed efficiency, and experienced fewer cases of diarrhea and fever. They also required fewer antibiotic treatments. Blood and liver analyses showed that GML enhanced antioxidant capacity, strengthened immune responses, and supported healthy liver metabolism. These findings suggest that GML helps calves better cope with early-life stress by improving both immunity and energy metabolism. Therefore, glycerol monolaurate may serve as a promising nutritional strategy to promote overall health status and reduce reliance on antibiotics in dairy production systems.

## 1. Introduction

The early growth stage of calves represents a critical period for their development, characterized by the rapid maturation of the digestive system, metabolic capacity, and immune function [1,2]. Nutritional management during this phase not only directly impacts animal health but also exerts lasting effects on long-term growth performance and productive capacity [3]. However, the transition from liquid to solid feed poses significant physiological challenges for calves: their digestive function is not yet fully developed, their gut microbiota is unstable, and their immune systems are still maturing. These factors render calves susceptible to gastrointestinal diseases, leading to reduced nutrient utilization efficiency and increased disease susceptibility, which in turn impacts animal welfare and production efficiency [2,4].

To address these challenges, antibiotics are commonly added to feed in conventional farming to maintain calf health. However, with the growing concern over antibiotic resistance and increasing consumer demand for antibiotic-free products, exploring sustainable alternatives has become a priority in veterinary and animal husbandry fields [5]. Medium-chain fatty acids (MCFAs) have well-documented benefits, including high absorption efficiency, antimicrobial activity, and the ability to regulate host metabolism and immune function [6]. Unlike long-chain fatty acids, MCFAs are efficiently utilized by young animals and exert antimicrobial effects by disrupting microbial cell membrane structures [7].

Glycerol monolaurate (GML), a monoglyceride of lauric acid, is a naturally occurring medium-chain fatty acid derivative found in coconut oil and milk fat, exhibiting broad-spectrum antibacterial, antiviral, and anti-inflammatory properties [8]. Numerous studies in monogastric animals have demonstrated that dietary supplementation with GML enhances intestinal barrier function, modulates gut microbiota, and regulates immune responses through multiple mechanisms. These include disrupting microbial cell membranes, interfering with quorum sensing, regulating immune signaling pathways, and boosting antioxidant capacity [6,9,10]. However, research on GML’s effects in ruminants remains limited.

Furthermore, given significant differences between ruminants and monogastric animals in digestive physiology, rumen development, and lipid metabolism, GML’s effects on calves may differ from those observed in monogastric animals [7,11]. Beyond the gut, GML may also influence hepatic immune and metabolic functions, which are crucial for maintaining physiological homeostasis during early calf development. The liver plays a critical role in nutrient metabolism, oxidative stress regulation, and inflammation control [4].

Therefore, this study proposes the following hypothesis: dietary supplementation with GML can improve gastrointestinal function in calves, enhance hepatic immune and metabolic activity, stabilize their physiological state, and thereby promote growth performance in Holstein calves. To validate this hypothesis, this study systematically evaluated calf growth performance and serum biochemical indicators. Multidimensional analyses were conducted, integrating liver transcriptomics and lipidomics with quantitative PCR validation. The findings aim to provide theoretical support for the application of GML as a functional fat additive in calf nutrition, advancing the development of antibiotic-free, sustainable dairy farming systems.

## 2. Materials and Methods

### 2.1. Animal Care and Use

All experimental procedures involving animals were approved by the Animal Research Ethics Committee of Northeast Agricultural University (Approval No. NEAUEC2024265). All animal handling and management practices were conducted in accordance with the institutional guidelines for the care and use of laboratory animals and complied with relevant national standards for animal welfare.

### 2.2. Source and Purity of Glycerol Monolaurate

Glycerol monolaurate (GML; purity ≥ 99%) used in this experiment was obtained from Hubei Lvxue Biological Industry Co., Ltd. (Xianning, Wuhan, China). The additive was stored under dry and dark conditions at room temperature and freshly mixed into the milk replacer prior to each feeding to ensure homogeneity and stability.

### 2.3. Animals, Experimental Design, and Diets

The animal trial was conducted between July and September 2023 at the Acheng Experimental Base of Northeast Agricultural University, located in Harbin, China (125°41′ E, 45°08′ N). A total of 24 Holstein bull calves were obtained from Modern Dairy Farming (Shuangcheng) Co., Ltd. (Harbin, China). Eligibility criteria included birth from multiparous dams with three previous parities. Throughout the experimental period, calves were routinely observed and managed by trained personnel. At the beginning of the trial, calves were 7 ± 0.5 days of age and had a mean body weight of 40.94 ± 4.93 kg, with no apparent differences in body size among individuals. Immediately after parturition, calves were fed pooled colostrum at a volume equivalent to 10% of body weight within the first 2 h of life, followed by an additional 2 L administered within the subsequent 12 h. Colostrum composition was analyzed using a MilkoScan 104 analyzer (Foss, Hillerød, Denmark). The results showed average concentrations (mean ± SD) of 17.82 ± 0.28% protein, 6.35 ± 0.18% fat, and 27.40 ± 0.05% total solids, with IgG concentrations exceeding 58 g/L. Calves were subsequently fed a commercial milk replacer manufactured by Honneur (Beijing, China), with a dry matter content of 94.34 ± 0.23% (dry matter basis: crude protein 22.31 ± 0.27%, crude fat 16.78 ± 0.19%, ash 6.87 ± 0.15%, and lactose 40.38 ± 0.11%). The ingredient composition and nutrient profile of the starter feed are presented in Table 1. Upon completion of the colostrum-feeding period, calves were housed individually in straw-bedded pens measuring 1.5 m × 1.2 m. Calves were stratified based on birth date and then randomly allocated to one of two dietary treatments, with 12 animals assigned to each group. The control group received a milk replacer without additives, whereas the treatment group was provided the same milk replacer supplemented with GML. The GML was freshly mixed into the milk replacer prior to feeding at a dosage of 100 mg per kg of body weight. Milk replacer was reconstituted daily at 135 g/L using boiled water cooled to feeding temperature, and calves were fed twice daily (08:00 and 18:00) following a step-up and step-down feeding schedule. Specifically, calves received 2 L per feeding from days 2 to 7, 3 L from days 8 to 14, 4 L from days 15 to 21, and 5 L from days 22 to 28. Thereafter, the volume was gradually reduced to 4 L from days 29 to 35, 3 L from days 36 to 40, 2 L from days 41 to 44, and 1 L on day 45. Based on the feeding schedule and a milk replacer solids concentration of 135 g/L, milk replacer dry matter intake ranged from approximately 0.54 to 1.35 kg/d across the study period and was identical for all calves regardless of treatment. All calves across all treatment groups consistently consumed their entire milk allowance at each feeding, and no refusals were observed. When averaged over the experimental periods (d 1–23 and d 23–45), milk replacer intake was comparable between phases due to the symmetrical step-up and step-down feeding design. For experimental consistency, day 7 after birth was designated as day 0. Starter feed was introduced on day 14 of the experiment, and individual feed intake as well as refusals were recorded on a daily basis. Calves were not weaned during the study period. The trial concluded on day 45, during which animals had ad libitum access to starter feed and fresh drinking water. Starter feed samples were collected at three time points (days 0–2, 14–16, and 28–30). Samples were initially dried at 60 °C for 48 h and subsequently oven-dried at 105 °C for 8 h to determine dry matter content. Dried samples were ground to pass through a 2 mm sieve and stored for subsequent analysis. Concentrations of dry matter (DM), crude protein (CP), ether extract (EE), and ash were determined according to standard AOAC International (2005) procedures (Official Methods of Analysis, 18th ed., AOAC International, Gaithersburg, MD, USA, 2005). Neutral detergent fiber (NDF) and acid detergent fiber (ADF) were analyzed using the method described by Van Soest et al., while starch content was quantified using a commercial Megazyme K-TSTA assay kit (Megazyme, Wicklow, Ireland).

### 2.4. Growth Performance and Nutrient Digestibility

Throughout the trial period, individual intake and refusal of calf starter were recorded daily. Body weight was measured on days 0, 23, and 45 before morning feeding using a digital platform scale. Average daily gain (ADG) was calculated as the average daily change in body weight over each period. Feed efficiency was derived by dividing ADG by the total consumption of milk solids and starter feed. On the same days, body length and heart girth were measured with a flexible ruler and a Biltmore stick (Jiangsu Animal Husbandry Veterinary Equipment Manufacturing Co., Ltd., Taizhou, China). During measurements, calves were maintained in a straight posture with limbs relaxed and naturally positioned, and care was taken not to pinch the tail. After the fecal samples collected on day 23 and day 45 of the experiment underwent the same drying and grinding procedures, the concentrations of DM, CP, EE, ash, NDF and ADF were all determined using the same detection method as the feed analysis (refer to AOAC International Standards, 2005; Van Soest et al.). Acid insoluble ash was used as an internal marker to estimate the apparent total-tract nutrient digestibility [12].

### 2.5. Health

Daily at 08:00, the staff recorded the rectal temperature of each calf and scored feces on a five-point scale ranging from normal to watery. A score of three or higher was defined as one day of diarrhea, and the total number of such days was used to calculate diarrhea incidence as an indicator of calf health during the trial. When signs of diarrhea were observed, treatment decisions were made individually based on clinical assessment. Cases requiring therapy received gentamicin, sodium ampicillin, and compound sulfonamides in combination with electrolyte solutions, with dosages adjusted according to individual body weight. Fever was defined as an abnormal elevation in rectal temperature, and affected calves were treated with penicillin and kanamycin following standard veterinary protocols. Antibiotic treatments were applied as part of routine farm health management based on clinical necessity; however, detailed records of treatment timing, duration, and exact dosages were not systematically maintained and therefore were not included in subsequent quantitative analyses. Fecal samples were collected by rectal swabbing at the scheduled time points of day 23 and 45 and preserved according to standardized procedures for subsequent analysis to assess digestive status.

### 2.6. Blood Collection and Analyses

Blood samples were collected from the jugular vein of calves on Days 0, 23, and 45 of the experiment (all prior to morning feeding). Blood collection utilized 10-milliliter centrifuge tubes containing sodium heparin and disposable needles. Immediately after collection, samples were centrifuged at 4 °C and 2000× *g* for 15 min. The separated plasma was aliquoted and stored at −80 °C for subsequent laboratory analysis to evaluate antioxidant capacity, biochemical characteristics, and immune-related indicators.

Antioxidant status was evaluated by measuring total antioxidant capacity (T-AOC), superoxide dismutase (SOD), malondialdehyde (MDA), glutathione peroxidase (GSH-Px), and catalase (CAT). All antioxidant parameters were measured using colorimetric methods (spectrophotometry) with a Plus 384 microplate reader (Thermo Fisher Scientific, Waltham, MA, USA). Biochemical parameters included total protein (TP), albumin (ALB), glucose (GLU), triglycerides (TG), cholesterol (CHOL), alanine aminotransferase (ALT), aspartate aminotransferase (AST), high-density lipoprotein (HDL), and low-density lipoprotein (LDL) were quantified using an automated veterinary biochemical analyzer (URIT-8021 AVet, URIT Medical Electronics Co., Ltd., Guilin, China). Globulin (GLB) concentration was calculated as the difference between total protein and albumin concentrations.

Immunological parameters included immunoglobulins (IgA, IgM, IgG), interferon-γ (IFN-γ), interleukin-2 (IL-2), Interleukin-4 (IL-4), Tumor Necrosis Factor-α (TNF-α), and Serum Amyloid A (SAA) were measured using enzyme-linked immunosorbent assay (ELISA). All antioxidant, biochemical, and immunological assay kits were purchased from Nanjing Jiancheng Bioengineering Institute (Nanjing, China).

### 2.7. RNA Extraction, Library Construction, and Sequencing

On day 45 of the experiment, six calves were randomly selected from each treatment group for liver biopsy. Local anesthesia was administered with 0.25–1.0% procaine hydrochloride solution, and liver tissue was collected by percutaneous puncture using sterile needles. Samples were rinsed in pre-cooled phosphate-buffered saline (PBS), divided into two portions, and stored at −80 °C either directly or after 24 h in RNA stabilization reagent for subsequent biochemical and transcriptomic analyses [13].

Total RNA was extracted from 12 liver samples (approximately 100 mg each) using TRIzol reagent (Thermo Fisher Scientific, Waltham, MA, USA). RNA purity and integrity were verified using a NanoDrop 2000 spectrophotometer and a Bioanalyzer 2100 system (Agilent Technologies, Santa Clara, CA, USA). Samples with RNA integrity number >7.0 were used for sequencing [14]. Libraries were prepared with the Illumina TruSeq RNA Sample Preparation Kit, size-selected (300 ± 50 bp), quantified with a Qubit 4.0 fluorometer, and sequenced on an Illumina NovaSeq 6000 platform (LC-Bio Technology, Hangzhou, China) to obtain 150 bp paired-end reads.

Raw reads were trimmed with Cutadapt and evaluated with FastQC. Clean reads were aligned to the Bos taurus reference genome using HISAT2, and transcript assembly and quantification were performed with StringTie and RSEM. Expression levels were normalized as FPKM and TPM. Differentially expressed genes were identified with DESeq2 using |fold change| ≥ 1.8 and *p* < 0.05, and functional enrichment was performed using KEGG analysis with Benjamini–Hochberg correction (adjusted *p* < 0.05).

Sequencing data has been uploaded to NCBI (PRJNA1371589).

### 2.8. Quantitative RT-PCR

The mRNA levels of radical S-adenosyl methionine domain containing 2 (RSAD2), interferon-induced protein with tetratricopeptide repeats 1 (IFIT1), cyclic GMP-AMP synthase (CGAS), interferon-stimulated gene 15 (ISG15), C-C motif chemokine ligand 8 (CCL8), MX dynamin like GTPase 1 (MX1) and MX dynamin like GTPase 2 (MX2) were quantified through quantitative real-time polymerase chain reaction. Hepatic RNA was isolated using the TRIzol reagent and subsequently synthesized into complementary DNA through reverse transcription. Amplification reactions employed primers listed in Table A1, following the supplier’s guidelines (Sagon Biotech, Shanghai, China). Thermal cycling parameters consisted of initial denaturation at 95 °C for 30 s, followed by 40 cycles of denaturation at 95 °C for 5 s and annealing/extension at 60 °C for 34 s. A final extension step was performed at 72 °C for 3 min.

Gene expression levels were measured using quantitative real-time PCR with β-actin as the housekeeping gene. The fold change in gene expression was calculated using the comparative 2^−ΔΔCt^ method, where the data were normalized to β-actin and then relative to the control group.

### 2.9. Lipid Extraction and UHPLC–MS/MS Analysis of Calf Liver

Approximately 50 ± 5 mg of frozen liver tissue was homogenized at −10 °C in 200 µL ice-cold 50% methanol with a 6 mm steel bead using a Wonbio-96c tissue crusher (Shanghai Wonbio Technology, Shanghai, China). After extraction with 600 µL ice-cold methyl tert-butyl ether, vortexting, 40 kHz sonication for 30 min at 5 °C, and centrifugation (13,000× *g*, 15 min, 4 °C), the upper phase was dried under nitrogen and reconstituted in 100 µL isopropanol:acetonitrile (1:1, *v*/*v*), briefly sonicated, vortexed, centrifuged again, and transferred for UHPLC-MS/MS; pooled QC aliquots (10–20 µL) were injected every 4–6 runs [15].

Lipids were separated on a Waters ACQUITY UPLC system with a CSH C18 column (100 × 2.1 mm, 1.7 µm; Waters, UK) at 40 °C using a 0.3 mL/min, 10 min gradient (30–100% solvent B; solvent A acetonitrile:water 6:4 with 10 mmol/L ammonium formate and 0.1% formic acid; solvent B isopropanol:acetonitrile 9:1 with the same additives). Mass spectra were acquired on a TripleTOF 6600 (SCIEX, Framingham, MA, USA) in positive and negative ESI modes (curtain gas 30 psi, ion source gases 60 psi, 500 °C, +5000 V/−4500 V) in IDA mode over *m*/*z* 50–2000, collecting the 12 most intense ions (4 s exclusion), with calibration every 20 samples and QC injections every 10 analyses.

Data were converted to mzXML and processed with XCMS, CAMERA, and metaX (R 4.0) for peak alignment, retention-time correction, and annotation against KEGG, HMDB, and in-house databases. Features present in >80% of samples were retained; missing values were imputed with the minimum intensity, followed by total-ion normalization, log_10_ transformation, and removal of variables with QC RSD > 30%. PCA and PLS-DA (metaX, ropls) supported multivariate evaluation, differential lipids were defined by VIP > 1, |fold change| > 1.2, and *p* < 0.05, and KEGG enrichment used hypergeometric testing with Benjamini–Hochberg correction (adjusted *p* < 0.05).

### 2.10. Statistical Analysis

All datasets were screened for normal distribution and outliers with the UNIVARIATE procedure in SAS 9.4 (SAS Institute Inc., Cary, NC, USA); observations flagged as outliers were checked against raw records before retention in the analysis. Growth performance variables recorded over time (body weight, body length, heart girth) were evaluated with the MIXED procedure, using day 0 values as covariates and an autoregressive [AR(1)] covariance structure selected by the lowest AIC/BIC; the model was *Y_ij_* = *µ* + *α_i_* + *B_j_* + *β* (*X_ij_* − $\bar{X}$ *_i_*) + *E_ij_*, where *α_i_* denotes treatment, *B_j_* the block defined by birth date, *β* the regression coefficient on the covariate, and *E_ij_* the residual term. Starter intake, average daily gain, and feed efficiency were analyzed with the GLM procedure (*Y_ij_* = *µ* + *T_i_* + *E_i_*), treating treatment as the fixed effect and reporting least-square means with pooled standard errors. Fecal scores collected repeatedly were modeled with MIXED, including treatment as a fixed effect, calf as a random effect (*U_i_*), and the same AR(1) covariance structure; the model was *Y_ij_* = *μ* + *α_i_* + *U_i_* + *ε_ij_*. Plasma measurements were analyzed with a repeated measures MIXED model incorporating fixed effects of treatment, block, and time, day 0 age as the covariate, calf nested within block as a random effect (*C_ij_*), and a compound symmetry covariance structure (*Y_ijk_* = *μ* + *α_i_* + *β_j_* + *T_k_* + *δ* (*X_ijk_* − $\bar{X}$ ) + *C_ij_* + *E_ijk_*), chosen by AIC/BIC comparison; least square means were adjusted for the covariate when reported. Statistical significance was declared at *p* ≤ 0.05, and tendencies (0.05 < *p* ≤ 0.10) were noted when relevant.

## 3. Results

### 3.1. GML Enhances Growth Performance in Calves, with Improved Nutrient Utilization

As shown in Table 2, GML supplementation did not significantly affect body weight, body length, or heart girth of calves at day 23. However, at day 45, body weight was significantly higher in the GML group than in the control group (*p* = 0.01). Calves receiving GML exhibited greater average daily gain during d 23–45 (*p* = 0.02) and over the entire experimental period (*p* = 0.02). Starter intake was significantly increased during d 23–45 (*p* = 0.02) and across the whole period (*p* = 0.03). Feed efficiency was significantly improved during d 1–23 (*p* = 0.01) and over the entire period (*p* = 0.03), but was not significantly affected during d 23–45.

These results indicate that GML supplementation improved the growth performance of calves, particularly during the later stage of the trial. To further elucidate the underlying factors contributing to this improvement, nutrient intake and total-tract apparent digestibility are presented in Table 3. As shown, GML supplementation did not affect nutrient intake at day 23, whereas DM, CP, and EE intake were all significantly increased at day 45 (*p* < 0.05). Apparent total-tract digestibility of DM was not affected by treatment. Crude protein digestibility was similar at day 23 but significantly increased at day 45 (*p* < 0.05), while ether extract digestibility was significantly greater in the GML group at both time points (*p* < 0.05). In contrast, NDF digestibility was not affected by treatment.

### 3.2. GML Supplementation Improves Fecal Consistency and Reduces Disease Incidence

As shown in Table 4, dietary supplementation with GML significantly improved several health-related parameters in calves. Calves in the experimental group had lower fecal scores during d 23–45 (*p* = 0.02), indicating improved fecal consistency. Additionally, the number of abnormal fecal days was markedly reduced during both d 23–45 (*p* = 0.001) and the overall period (*p* = 0.004) compared with the control group. The incidence of diarrhea also decreased significantly during d 23–45 (*p* = 0.043) and across the entire experimental period (*p* = 0.013). Furthermore, the number of fever cases was significantly reduced during d 23–45 (*p* < 0.001), and calves in the experimental group required fewer antibiotic treatments during the same period (*p* = 0.017). Collectively, these findings demonstrate that GML supplementation effectively alleviated gastrointestinal disturbances and reduced disease incidence, thereby contributing to improved health and reduced dependence on antibiotic intervention in calves.

### 3.3. Effects of GML on Plasma Biomarkers

As presented in Table 5, there were no significant differences in plasma concentrations of ALB, AST, LDH, IgM, IL-4, and SAA between the two treatments. However, calves in the GML group showed significantly higher plasma concentrations of GLU (*p* = 0.01), GLB (*p* = 0.04), GSH-Px (*p* = 0.02), and IgG (*p* = 0.02), as well as elevated CAT activity (*p* = 0.02), compared with those in the Control group. In contrast, the plasma concentrations of ALP (*p* = 0.03), ALT (*p* = 0.04), IL-2 (*p* = 0.03), and IFN-γ (*p* = 0.03) were significantly reduced in GML calves. Moreover, T-AOC activity was markedly elevated (*p* = 0.02) in the GML group relative to the Control group, indicating enhanced antioxidant capacity following GML supplementation.

### 3.4. Integrated Transcriptome Profiling and qPCR Validation Reveal Hepatic Benefits of GML

**Figure 1:**
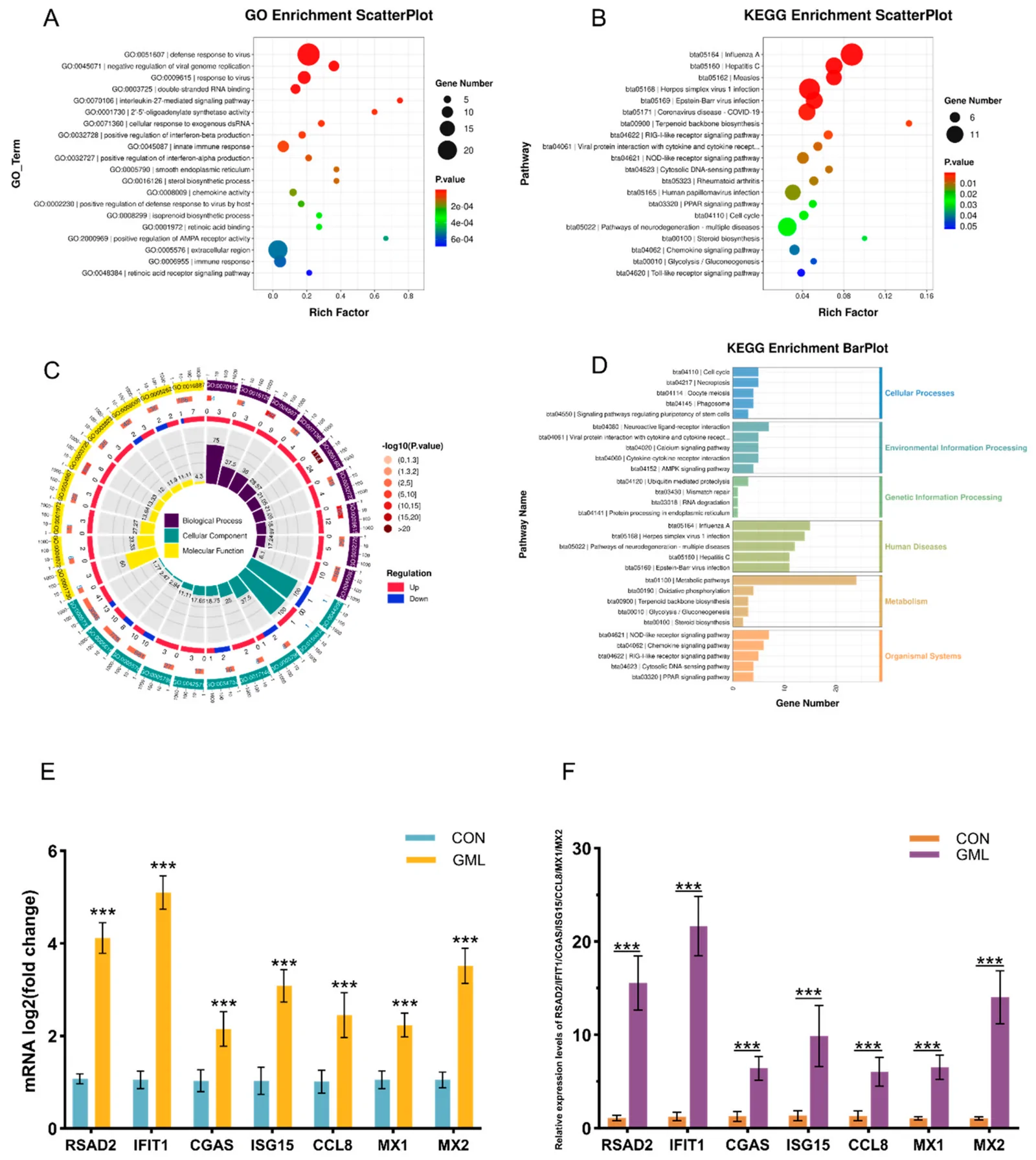
Transcriptomic discovery and qPCR validation reveal the impact of GML supplementation on liver metabolism in calves. (**A**) Gene Ontology (GO) enrichment bubble plot showing the top enriched biological processes of differentially expressed genes (DEGs). (**B**) Kyoto Encyclopedia of Genes and Genomes (KEGG) enrichment bubble plot illustrating significantly enriched pathways associated with GML treatment. (**C**) Circular plot displaying the proportion of DEGs involved in major functional pathways. (**D**) Functional classification of DEGs based on KEGG pathway categories. (**E**) Identification of differentially expressed genes (DEGs) related to immune regulation and lipid metabolism from RNA-seq analysis in GML-supplemented calves. (**F**) Quantitative real-time PCR (qPCR) validation of selected genes related to immune regulation and lipid metabolism (RSAD2, IFIT1, CGAS, ISG15, CCL8, MX1, MX2, *** *p* < 0.001).

In order to fully analyze the differences in transcriptomic changes between the two groups, we carried out transcriptome sequencing on 12 samples. We obtained an average of 60,777,648.67 raw reads per sample. After filtering with SeqPrep and Sickle, the average high-quality reads reached 59,665,523 about 98%, and all samples achieved Q30 scores exceeding 95%, confirming data reliability. The DESeq2 analysis of the 12,788 genes resulted in the identification of 345 differentially expressed genes (DEGs), of which 219 were upregulated and 126 downregulated. GO enrichment analysis (Figure 1A) revealed multiple biological processes such as RNA splicing, ribosome assembly, guanine nucleotide exchange factor activity and others. KEGG pathway analysis (Figure 1B–D) indicated that nine pathways were significantly enriched including genetic information processing (base excision repair, proteasome), metabolism (histidine and fructose metabolism) and immune response (complement and coagulation cascades). In addition, qPCR validation (Figure 1F) indicated significant differences in expression (*p* < 0.001) between GML and control groups for the given genes, consistent with RNA-seq findings (Figure 1E), which demonstrates that GML feeding affects liver lipid metabolism, immune response, inflammation relief.

As illustrated in Figure 1F, the expression of relative mRNA expression of selected genes were confirmed in qPCR, which validated the transcriptomic results, and showed that expression levels of all the target genes were different (*p* < 0.001) between the control and the GML-supplemented group. In particular, the genes involved in lipid metabolism and immune regulation were up-regulated while those involved in inflammation responses were significantly down-regulated in the GML group. In accordance with a consistent change direction between qPCR and RNA-Seq data, the sequencing data is generally reliable and it suggests that GML supplementation significantly modifies gene expression in the liver and enhances hepatic metabolism and reduces hepatic inflammation in calves.

### 3.5. GML Induces Targeted Modulation of the Lipid Metabolome

**Figure 2:**
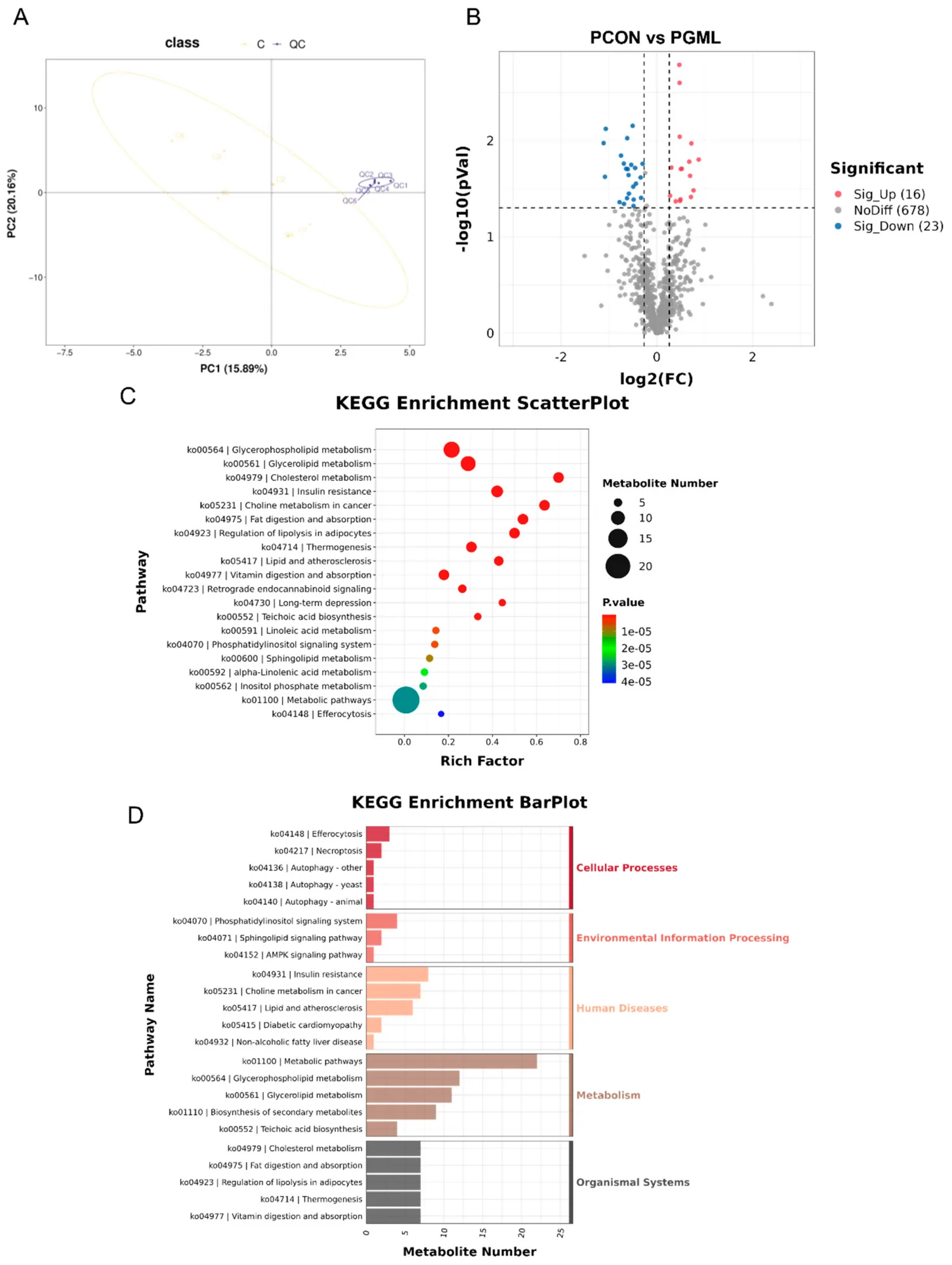
Lipidomic profiling in GML-supplemented calves. (**A**): Partial least squares–discriminant analysis (PLS-DA) scores plot showing clear group separation and tight within-group clustering. (**B**): Volcano plot of lipid features, highlighting 39 differentially abundant lipids between GML and control groups. (**C**): KEGG enrichment bubble plot of significantly affected lipid metabolism pathways. (**D**): KEGG pathway classification of differential lipids highlighting major metabolic categories.

In the lipidomics dataset, PLS-DA clustered tightly the GML and CON samples showing generally similar overall lipid profiles (Figure 2A) and also permtest showed no overfitting, since the R2 curve is above the Q2 curve with negative Q2 intercept. The differential analysis among 717 lipid metabolites highlighted 39 features modified in GML relative to CON (16 up and 23 down), as can be seen in the volcano plot (Figure 2B). Remarkably, several upregulated lipids were intermediates and storage forms of energy, such as diglyceride DG 16:0_18:2 and triglycerides TG 16:0_18:1_18:1 and TG 16:0_18:1_18:2, indicating increased lipid absorption or turnover (Table 6). In contrast, multiple structurally significant and signaling lipids were downregulated, including the polyunsaturated phospholipid PC 18:2_20:5, the ether-linked phospholipid PE O-22:5_16:1, and the sphingolipids SM 34:1;2O and SHexCer 41:4;3O, possibly indicative of change in the membranes and in their inflammatory signaling (Table 6). Linking up with the pathway diagram, and the end summary table, these differential lipids provide coherence of shifts around the related pathways of lipid metabolism and coherent shifts among nodes involved in fatty acid turnover, membrane lipid remodeling, and in the homeostasis of signaling lipids (Figure 2C,D). Combined, the data support the idea that in nursing calves, GML addition selectively influences a pathway-based control of the lipid metabolome, with a general consensus of the difference in global lipidomics between groups.

## 4. Discussion

The early postnatal stage is widely regarded as one of the most vulnerable periods in the development of dairy calves, as it is characterized by rapid physiological transitions and substantial environmental pressures. Calves’ immune system and gut are in their immature forms at early postnatal life; thus, their system is extremely vulnerable to infection. However, during the same period, massive and fast growth necessitates enough nutritional supply and effective metabolic maturation as well. Consequently, nutritional interventions for supporting both immune and metabolic maturation of the calves are most important. Medium-chain fatty acids, including lauric acid and its monoglyceride GML, have drawn particular attention in this context because of their rapid digestion, antimicrobial activity, and beneficial effects on intestinal and metabolic stability. Hence, we show that supplementing GML to milk formula, an early-life nutritional intervention, enhances calves’ health resilience and growth performance by inducing coordinated physiological responses. Significant improvements in weight gain, feed efficiency, and gut health, to cite a few, clearly indicate that GML may help calves navigate the delicate early developmental period more smoothly, thereby laying a solid healthy foundation for subsequent growth and development.

Regarding growth efficiency, the growth-promoting effects of GML supplementation in calves were evident throughout the experimental period, with improved feed utilization observed from the early stage onward. This early response likely reflects the rapid absorption and direct metabolic utilization of medium-chain fatty acids, which bypass extensive digestive processing and provide readily available energy. As starter intake increased during the later phase, these effects were amplified, resulting in greater body weight gain, ADG, and starter intake [10,16]. The progressive enhancement of growth performance across the trial period aligns with previous reports attributing such responses to gradual improvements in intestinal function and metabolic capacity [17].

In the present study, improvements in fecal consistency, fewer days with abnormal feces, and a lower incidence of diarrhea were observed in GML-supplemented calves, consistent with a potential beneficial effect on intestinal health. These observations are consistent with the known biological properties of lauric acid, the primary metabolite of GML. In weaned piglets, dietary lauric acid has been reported to influence intestinal microbiota composition, improve villus morphology, and reduce diarrhea [10,18]. Comparable effects have also been described in broiler chickens, where medium-chain fatty acids or their monoglycerides improved feed efficiency and growth performance, partly through enhanced lipid digestion and antimicrobial activity against enteric pathogens such as Clostridium and Salmonella [19]. These findings across species lead us to speculate that GML might play a role in supporting intestinal barrier function and reducing pathogenic pressure during early-life feeding transitions, potentially influencing the efficiency with which specific nutrients are digested and utilized. To further evaluate nutrient utilization, calculated nutrient intake and total-tract apparent digestibility were assessed. GML supplementation increased nutrient intake during the later stage, yet did not affect the apparent digestibility of DM, NDF, or ADF at either sampling period, suggesting that the observed benefits were likely driven primarily by enhanced feed intake coupled with selective improvements in nutrient utilization rather than a generalized enhancement of digestive capacity. The selective digestibility response was dependent on nutrient type and developmental stage: apparent digestibility of EE was higher in GML-supplemented calves as early as day 23 and remained elevated at day 45, consistent with the rapid absorption and metabolism of medium-chain fatty acids [20]. In contrast, the improvement in CP digestibility was observed only at day 45, coinciding with increased starter intake and more advanced digestive development, indicating that protein utilization may be more responsive to GML supplementation as the digestive system matures [21].

Improved intestinal condition is likely to contribute to more efficient nutrient utilization. By reducing subclinical intestinal disturbances, GML supplementation may facilitate nutrient absorption from both milk replacer and starter feeds, thereby improving feed efficiency. In line with this, higher plasma glucose concentrations were observed in GML-supplemented calves, indicating enhanced systemic energy availability [7]. This response may be attributed to a combination of improved intestinal absorption and altered hepatic energy metabolism [22]. Medium-chain fatty acids are rapidly absorbed and metabolized, providing an efficient energy source without extensive digestive processing, which may favor nutrient partitioning toward growth rather than maintenance or inflammatory demands [23,24].

In addition to the improvements in intestinal condition and energy utilization discussed above, GML supplementation was associated with changes in several systemic health-related indicators. Calves receiving GML exhibited a lower incidence of fever, reduced antibiotic use, and a decreased prevalence of both intestinal and extra-intestinal pathogens, suggesting that local intestinal improvements may be linked to broader physiological responses [19]. Analysis of blood biochemical parameters showed reduced circulating IL-2 levels together with increased IgG concentrations, indicating a modulation of immune activity rather than a generalized immune activation [25]. In parallel, liver transcriptomic analysis revealed upregulation of genes involved in antiviral responses, which may reflect an enhanced state of innate immune readiness in young calves facing common viral challenges [26].

The plasma metabolite profiles indicate that GML exerts systemic effects that enhance metabolic efficiency, antioxidant defense, and immune modulation in suckling calves [27]. Significant reductions in plasma ALT and ALP activities suggest alleviation of hepatic and systemic metabolic stress [28]. ALT, a marker of hepatocellular integrity, and ALP, which reflects intestinal and bone metabolic turnover, both indicate that GML improves metabolic balance [29]. These reductions are likely a consequence of improved gastrointestinal health, as demonstrated by lower fecal scores, fewer abnormal fecal days, and decreased diarrhea incidence in GML-supplemented calves [30]. Although improvements in fecal consistency and reduced diarrhea incidence suggest a beneficial effect of GML on intestinal health, the underlying mechanisms were not directly assessed in this study. It is therefore speculative to propose that GML may influence intestinal barrier integrity, microbial translocation, or endotoxin exposure. Future studies incorporating direct measurements (e.g., intestinal permeability, circulating LPS, and microbiota composition) are required to validate these hypotheses [31]. This is reflected in the significantly improved growth rate and feed efficiency observed during the later stages of the trial. Furthermore, GML supplementation significantly enhanced systemic antioxidant capacity, as reflected by increased activities of CAT, GSH-Px, and T-AOC–findings consistent with previous reports in young animals [32,33]. The reinforced antioxidant defense is particularly beneficial during the early-life stage, when calves are susceptible to redox imbalance due to high metabolic demands and evolving immune challenges [34]. By ameliorating systemic oxidative stress, GML helps maintain intestinal epithelial integrity and supports overall gut health, which may explain the observed reduction in diarrhea incidence [18,35]. Moreover, this improvement in antioxidant status helps mitigate systemic oxidative stress, which can otherwise impair immune function and growth. These findings are consistent with previous studies in other species, such as broiler chickens, where dietary lauric acid improved antioxidant enzyme activity and reduced lipid peroxidation [17,36]. In terms of immune modulation, GML significantly reduced plasma interleukin-2 (IL-2), indicating controlled T-cell activation and reduced inflammation [37]. This anti-inflammatory effect, however, was balanced by an increase in GLB and IgG, indicating enhanced humoral immunity rather than compromised immune competence [38]. This optimized immune response likely explains the reduced incidence of fever and antibiotic use. It should be noted that health management interventions were conducted according to routine farm veterinary practices throughout the trial, which may have contributed to variability in physiological responses among individuals. The upregulation of interferon-stimulated genes (RSAD2, MX1) in liver tissue further suggests that GML primes innate antiviral defenses while tempering excessive adaptive immune activation, offering a dual mechanism for improving health outcomes [39,40].

Integrated transcriptomic and lipidomic analyses revealed immunometabolic alterations associated with GML supplementation. Dietary supplementation with GML has been shown to improve growth performance and health in early-life calves. In this study, we conducted integrated transcriptomic analysis, including RNA-seq, qPCR validation, GO analysis, and KEGG pathway enrichment, to investigate the underlying molecular mechanisms. The main finding is that GML induces extensive transcriptional reprogramming in the liver, with strong upregulation of genes associated with the innate immune response, establishing a pre-activated antiviral state. The integrated transcriptomic analysis indicates that GML supplementation induces immunometabolic reprogramming in the liver of calves, primarily characterized by a potent and coordinated activation of antiviral defenses. Significant enrichment of KEGG pathways related to viral infections—such as Influenza A, Hepatitis C, and Herpes simplex—does not reflect active infection but indicates the priming of core intracellular defense mechanisms common to diverse viral challenges [41,42]. This interpretation is supported by GO analysis, which showed upregulation of biological processes including “defense response to virus” and “negative regulation of viral genome replication” [43]. Enhanced viral recognition is suggested by the enrichment of the molecular function “double-stranded RNA binding,” pointing to the involvement of cytosolic sensors such as RIG-I and MDA5 [44,45]. Upon activation, these receptors trigger signaling cascades that induce type I interferons (IFN-α/β), which in turn drive the expression of a suite of interferon-stimulated genes (ISGs) [46]. Functional validation by qPCR confirmed strong upregulation of key antiviral ISGs [47], including RSAD2, IFIT1, MX1, MX2, ISG15, and CCL8. These genes encode effector proteins with diverse antiviral roles: MX1 and MX2 inhibit viral nucleocapsid transport, IFIT1 sequesters viral RNA, RSAD2 disrupts viral membrane integrity and generates antiviral nucleotides, and ISG15 modulates protein function via ISGylation [48]. Collectively, these effectors establish a broad-spectrum intracellular environment that restricts viral replication. In summary, GML acts as an effective innate immunomodulator by pre-activating the type I interferon pathway and its downstream ISG network. This transcriptional pattern was associated with a capacity for faster and stronger antiviral responses, providing a molecular explanation for the observed reductions in clinical signs such as diarrhea, fever, and antibiotic use, and underscoring the value of GML in enhancing disease resilience during early life.

Furthermore, Lipidomic profiling provides a coherent picture of hepatic metabolic reprogramming that complements the transcriptomic evidence of immune activation in GML-supplemented calves. The observed lipid shifts are not isolated changes; rather, they represent a functionally integrated adaptation likely orchestrated to support the energetic and structural demands of an activated immune system while simultaneously modulating inflammatory tone. Specifically, the accumulation of diacylglycerol (DG 16:0_18:2) and triacylglycerol (TG 16:0_18:1_18:1, TG 16:0_18:1_18:2) species points to an anabolic restructuring of hepatic neutral lipid metabolism [49,50]. Alternatively, such accumulation may reflect adaptive lipid storage or transient metabolic stress in response to increased metabolic demands. This remodeling likely serves dual purposes: it creates a readily mobilizable energy reservoir to fuel the high metabolic costs of synthesizing antiviral effector proteins and immune cells, a process supported by elevated plasma glucose levels [51,52,53]. Furthermore, DGs not only serve as TG precursors but also act as signaling lipids. The accumulation of DG 16:0_18:2 may influence protein kinase C–mediated signaling pathways, which intersect with immune cell activation and metabolic regulation, an area that warrants further investigation [54,55]. Concurrently, alterations in glycerophospholipid species, particularly the reduction in PC 18:2_20:5 and PE O-22:5_16:1, suggest strategic remodeling of membrane lipid architecture [56]. Polyunsaturated fatty acid (PUFA)–containing phospholipids are essential for maintaining membrane fluidity and serve as precursors for eicosanoid signaling molecules [57,58]. The decrease in these highly unsaturated species may reflect a metabolic shift to limit the production of pro-inflammatory eicosanoids derived from arachidonic acid (20:4) or eicosapentaenoic acid (20:5), contributing to the controlled inflammatory state evidenced by reduced plasma IL-2 [59,60]. This remodeling may also facilitate the formation of more ordered lipid rafts, which serve as platforms for organizing innate immune signaling complexes, including those involved in the interferon response pathway highlighted by our transcriptomic data [61]. Additionally, the reduction in sphingolipids, such as sphingomyelin (SM 34:1;2O) and sulfatide (SHexCer 41:4;3O), provides a molecular link to improved systemic health [62]. Sphingolipids, particularly ceramide, are key regulators of insulin resistance and pro-inflammatory apoptosis [63]. A reduction in ceramide derivatives, such as SM and sulfatide, likely signals a downregulation of this detrimental signaling axis, which aligns with the observed systemic benefits of improved metabolic efficiency and a controlled inflammatory response [64]. This modulation is consistent with findings in rodent models in which lauric acid alleviated metabolic inflammation through sphingolipid metabolism [65]. Together, these transcriptomic and lipidomic alterations suggest that GML shapes a more balanced immunometabolic state in the liver, allowing calves to respond to early-life challenges with improved efficiency. This coordinated adjustment provides a mechanistic context for the enhanced health and growth observed in GML-supplemented calves.

## 5. Conclusions

In conclusion, dietary supplementation with glycerol monolaurate is associated with enhanced growth and improved health in calves. These improvements are linked to alterations in hepatic immunometabolic profiles, as revealed by integrated transcriptomic and lipidomic analyses. GML was associated with a pre-activated antiviral state in the liver and restructured lipid metabolism, which may support energy needs and help modulate inflammation. These findings suggest that GML may serve as a viable functional additive for promoting resilience and metabolic efficiency, offering a sustainable strategy to reduce antibiotic use in calf production. However, the study’s limitations include the short duration of the trial and the absence of long-term data. Future research should explore the long-term impacts of GML, particularly its role in supporting immune maturation across different environmental and management conditions.

## Figures and Tables

**Table 1:** Feed ration formula and nutrient levels.

Nutrient Levels	Content
Ingredient	
Ground corn	39
Extruded soybean	9.5
DDGS	4
Soybean meal	27
Soybean hulls	6.6
Wheat bran	6.6
Molasses	3
Limestone	1.5
Sodium bicarbonate	0.4
Sodium chloride	0.8
Magnesium oxide	0.2
Calcium hydrogen phosphate	0.4
Premix ^1^	1
Chemical composition	
DM (%)	89.3
CP (%DM)	24.0
NDF (%DM)	18.1
ADF (%DM)	8.78
Ash (%DM)	7.5
EE (%DM)	4.7
Starch (% DM)	32

^1^ Premix supplied the following nutrients per kilogram of mixed feed: vitamin A 6500 IU, vitamin D 2000 IU, vitamin E 120 IU, Fe 80 mg, Co 0.3 mg, Cu 16 mg, I 1.1 mg, Mn 50 mg, Se 0.45 mg, and Zn 70 mg.

**Table 2:** Effect of GML on growth performance of calves.

Items	Trial Period (d)	Treatment ^1^		SEM	*p*-Value
		Control	GML		
Body weight (kg)	23	57.53	59.82	1.335	0.26
	45	68.51	76.24	1.933	0.01
Body length (cm)	23	80.11	82.34	2.296	0.48
	45	84.33	86.22	1.466	0.38
Heart girth (cm)	23	87.89	85.67	1.11	0.18
	45	100.56	99.89	1.767	0.79
Starter intake (g/d)	1–23	95.6	97.2	10.84	0.91
	23–45	781.6	850.4	22.06	0.02
	1–45	438.6	473.8	20.96	0.03
Average daily gain (kg/d)	1–23	0.62	0.77	0.040	0.09
	23–45	0.80	0.85	0.053	0.02
	1–45	0.71	0.81	0.047	0.02
Feed conversion ratio ^2^	1–23	1.72	1.39	0.036	0.01
	23–45	1.54	1.54	0.024	0.32
	1–45	1.64	1.47	0.026	0.03

^1^ Control: calves received a conventional diet with no additive; GML: calves received a conventional diet and GML (100 mg/kg of BW). ^2^ Feed conversion ratio = (milk solids and starter intake)/ADG.

**Table 3:** Nutrient intake and total-tract apparent digestibility of dairy calves fed GML.

Items	Trial Period (d)	Treatment		SEM	*p*-Value
		Control	GML		
Milk Replacer Intake (kg/d)					
DM	23	1.35	1.35	-	-
	45	0.27	0.27	-	-
CP	23	0.30	0.30	-	-
	45	0.06	0.06	-	-
EE	23	0.23	0.23	-	-
	45	0.05	0.05	-	-
Starter Intake (kg/d)					
DM	23	0.22	0.23	0.015	0.89
	45	0.84	0.91	0.048	0.01
CP	23	0.05	0.05	0.004	0.91
	45	0.20	0.22	0.012	0.04
EE	23	0.03	0.03	0.003	0.87
	45	0.04	0.05	0.004	0.04
NDF	23	0.01	0.01	0.001	0.76
	45	0.14	0.15	0.011	0.06
Nutrient Apparent Digestibility (%)					
DM	23	73.58	73.94	0.063	0.76
	45	80.00	80.01	0.197	0.79
CP	23	71.99	71.68	0.307	0.42
	45	72.7	73.74	0.244	0.01
EE	23	77.78	79.05	0.142	0.01
	45	79.10	79.98	0.054	0.03
NDF	23	48.8	48.84	1.387	0.97
	45	46.52	44.59	1.686	0.43

**Table 4:** Effects of GML supplementation on health parameters in calves.

Items	Trial Period (d)	Treatment		SEM	*p*-Value
		Control	GML		
Fecal score	1–23	1.66	1.49	0.059	0.77
	23–45	1.46	1.28	0.032	0.02
	1–45	1.56	1.39	0.044	0.36
Abnormal fecal days (d)	1–23	5.11	4.44	0.345	0.19
	23–45	3.78	1.89	0.395	0.001
	1–45	8.89	6.33	0.448	0.004
Diarrhea case (times)	1–23	2.08	1.31	0.308	0.09
	23–45	1.46	0.85	0.204	0.04
	1–45	1.77	1.08	0.189	0.01
Number of fevers	1–23	1.00	1.58	0.281	0.16
	23–45	1.07	0.20	0.132	<0.001
	1–45	1.04	0.81	0.171	0.36
Treated with antibiotic times (times)	1–23	1.43	1.87	0.189	0.11
	23–45	1.76	1.12	0.184	0.02
	1–45	1.63	1.43	0.138	0.31

**Table 5:** Effect of GML on plasma metabolites in Holstein calves.

Items	Treatment		SEM	*p*-Value ^1^		
	Control	GML		Tri	Time	Tri × Time
Biochemical levels						
GLU (mmol/L)	5.36	5.75	0.102	0.01	0.07	0.47
TP (g/L)	63.16	63.71	0.549	0.49	0.005	0.23
ALB (g/L)	31.11	31.55	0.428	0.47	0.003	0.55
GLB (g/L)	33.16	36.07	0.941	0.04	<0.001	<0.001
ALT (IU/L)	10.09	9.75	0.110	0.04	<0.001	<0.001
AST (IU/L)	45.72	45.60	0.612	0.89	<0.001	0.61
ALP (IU/L)	182.50	171.00	3.337	0.03	<0.001	0.002
LDH (IU/L)	1012.50	1056.50	22.427	0.18	<0.001	<0.001
Antioxidant levels						
T-AOC (U/mL)	9.01	9.58	0.156	0.02	0.003	0.47
CAT (U/mL)	42.48	45.83	0.912	0.02	0.005	0.39
GSH-Px (U/mL)	368.62	378.89	3.075	0.02	<0.001	0.99
MDA (nmol/mL)	4.16	3.93	0.093	0.10	0.43	0.91
SOD (U/mL)	74.12	73.23	1.236	0.62	<0.001	0.38
Immunological levels						
IgA (g/L)	1.72	1.76	0.021	0.25	0.08	0.70
IgM (g/L)	0.98	0.99	0.010	0.71	0.51	0.01
IgG (g/L)	2.18	2.25	0.022	0.02	0.30	0.60
TNF-α (pg/mL)	278.82	283.41	2.929	0.28	0.57	0.64
IL-2 (pg/mL)	48.04	44.10	1.238	0.03	0.009	0.22
IL-4 (ng/L)	90.77	90.56	1.431	0.87	0.35	0.59
IFN-γ (pg/mL)	1.01	0.97	0.124	0.03	0.06	0.18
SAA (μg/mL)	902.85	888.91	2.929	0.28	0.57	0.64

^1^ *p*-value: Tri = treatment effect; Time = time effect; Tri × Time = interaction between treatment and time effect.

**Table 6:** Identification of significantly different metabolites between the CON group and GML group.

KEGG Pathway ID	KEGG Pathway Name	Metabolite ID	Metabolite Name	*p*-Value	VIP	CON vs. GML
ko00561	Glycerolipid metabolism	C00641	DG 16:0_18:2	0.03	1.1132	up
		C00422	TG 16:0_18:1_18:1	0.005	1.7616	up
		C00422	TG 16:0_18:1_18:2	0.01	1.6457	up
ko00564	Glycerophospholipid metabolism	C00157	PC 18:2_20:5	0.02	1.2399	down
		C04475	PE O-22:5_16:1	0.03	1.9906	down
ko00600	Sphingolipid metabolism	C00550	SM 34:1;2O	0.01	1.1453	down
		C06125	SHexCer 41:4;3O	0.004	1.1941	down

## Data Availability

The data presented in this study are available on request from the corresponding author. The data are not publicly available due to privacy and ethical restrictions.

## Appendix A

**Table A1:** Sequences of primers used for RT-PCR.

Primer	Sequence
β-actin F	CGTGACATCAAGGAGAAGCT
β-actin R	CTAGAAGCATTTGCGGTGGA
RSAD2 F	AGGAACAGATAACCGCGCTC
RSAD2 R	CACATCCTTGTGGCGATCCA
IFIT1 F	GCCTCAAGTAGAAAAGCAATCCC
IFIT1 R	CATGTAGAGGCATGTATGTTCTGA
CGAS F	AAGGCCTGCCCATCAGTAAC
CGAS R	GACAGCCGCCATGTTTCTTC
ISG15 F	GGTATCCGAGCTGAAGCAGTT
ISG15 R	ACCTCCCTGCTGTCAAGGT
CCL8 F	GCGAGAAGAGCCCTGATTGTACTG
CCL8 R	AGCCTCTACAGCAGCCTCCTTC
MX1 F	GTACGAGCCGAGTTCTCCAA
MX1 R	ATGTCCACAGCAGGCTCTTC
MX2 F	CTTCAGAGACGCCTCAGTCG
MX2 R	TGAAGCAGCCAGGAATAGTG
